# Spatiotemporal Changes in *Plasmodium vivax msp1_42_* Haplotypes in Southern Mexico: From the Control to the Pre-Elimination Phase

**DOI:** 10.3390/microorganisms10010186

**Published:** 2022-01-15

**Authors:** Alejandro Flores-Alanis, Lilia González-Cerón, Frida Santillán-Valenzuela, Cecilia Ximenez, Marco A. Sandoval-Bautista, Rene Cerritos

**Affiliations:** 1 Departamento de Microbiología y Parasitología, Facultad de Medicina, Universidad Nacional Autónoma de México (UNAM), Mexico City 04360, Mexico; bioalejandrofa@gmail.com; 2 Regional Center for Public Health Research, National Institute of Public Health (INSP), Tapachula 30700, Mexico; fsantill@insp.mx (F.S.-V.); masb@insp.mx (M.A.S.-B.); 3 Unidad de Medicina Experimental, Facultad de Medicina, Universidad Nacional Autónoma de México (UNAM), Mexico City 06720, Mexico; cximenez@unam.mx; 4 Centro de Investigación en Políticas, Población y Salud, Facultad de Medicina, Universidad Nacional Autónoma de México (UNAM), Mexico City 04510, Mexico

**Keywords:** *Plasmodium vivax*, merozoite surface protein 1 (42 kD), *pvmsp1_42_*, haplotype diversity, haplogroups, B-cell epitopes, control and pre-elimination phases, southern Mexico

## Abstract

For 20 years, *Plasmodium vivax* has been the only prevalent malaria species in Mexico, and cases have declined significantly and continuously. Spatiotemporal genetic studies can be helpful for understanding parasite dynamics and developing strategies to weaken malaria transmission, thus facilitating the elimination of the parasite. The aim of the current contribution was to analyze *P. vivax*-infected blood samples from patients in southern Mexico during the control (1993–2007) and pre-elimination phases (2008–2011). Nucleotide and haplotype changes in the *pvmsp1_42_* fragment were evaluated over time. The majority of multiple genotype infections occurred in the 1990s, when the 198 single nucleotide sequences exhibited 57 segregating sites, 64 mutations, and 17 haplotypes. Nucleotide and genetic diversity parameters showed subtle fluctuations from across time, in contrast to the reduced haplotype diversity and the increase in the R^2^ index and Tajima’s D value from 2008 to 2011. The haplotype network consisted of four haplogroups, the geographical distribution of which varied slightly over time. Haplogroup-specific B-cell epitopes were predicted. Since only high-frequency and divergent haplotypes persisted, there was a contraction of the parasite population. Given that 84% of haplotypes were exclusive to Mesoamerica, *P. vivax* flow is likely circumscribed to this region, representing important information for parasite surveillance.

## 1. Introduction

There are approximately 241 million malaria cases per year worldwide and 627,000 related deaths, according to the estimates of the World Health Organization (WHO) [1]. In Mexico, the number of cases fluctuated between 20,000 and 130,000 per year in the 1980s. In the following decade, the anti-malaria measures of the intensified control programs caused a gradual decrease in cases.

Anti-malarial control measures are implemented when the burden of malaria infection becomes an important public health problem and the slide positivity rate of fever cases is above 5%. The latter rate is a method for analyzing changes in malaria incidence; if it drops below 5% and certain other requirements are met, a country may enter the pre-elimination phase, which consists of the evaluation and reorientation of anti-malaria policies and strategies with the aim of continually reducing the number of cases [2]. Mexico has remained in the pre-elimination phase since 2007.

In Mexico, the number of malaria cases had declined to 514 by 2016 [3]. Nevertheless, in 2017 this country reported a 37% rise in cases—most notably in the states of Chiapas, Chihuahua, and Tabasco. Alarmingly, new cases began to appear in San Luis Potosi in the same year, a state formerly enjoying an absence of malaria transmission [1,3,4].

The multiple mosquito vectors implicit in *Plasmodium vivax* genetic and haplotype diversity contribute to successful transmission in distinct environments [5]. Such diversity potentially plays an important role in the development of parasite mechanisms for evasion of the vertebrate and invertebrate host immune response (even after the application of vaccines) and for the selection of strains resistant to current drugs [6,7]. Various evolutionary forces act on genes that code for blood-stage antigens, resulting in high polymorphism and parasite diversity. Thus, it is necessary to distinguish between the different species of parasites and trace their haplotypes [8]. Additionally, this information is essential for designing effective vaccines and surveillance strategies [9]. For countries moving to the elimination phase, therefore, a key strategy is the use of genetic studies with polymorphic markers to understand transmission dynamics. They provide evidence of the weakening of parasite transmission when the number of cases is diminishing, and aid in molecular surveillance.

The *P. vivax* merozoite surface protein 1 (PvMSP1) is a 200 kD protein coded by a gene located on chromosome 7 [10]. It is abundant on the surface of merozoites and, during invasion, makes the first contact with human reticulocytes [11]. The carboxyl end of the 42 kDa peptide (PvMSP1_42_) is processed into two segments of 33 and 19 kDa (PvMSP1_33_ and PvMSP1_19_, respectively). PvMSP1_19_ is formed by two highly conserved epidermal growth factor-like domains that bind to reticulocytes [12]. According to immuno-epidemiological studies, PvMSP1_33_ [13,14] and PvMSP1_19_ [15,16,17] are highly immunogenic and, consequently, could possibly be useful as vaccine candidates for *P. vivax*. However, a polymorphic segment of PvMSP1_33_ is presumably involved in the evasion of the host antibody response, as suggested by evidence that PvMSP1_33_ might be under balancing selection [18,19,20,21].

In the current contribution, an analysis was made of the nucleotide and haplotype diversity of *P. vivax* *msp1_42_*, considering temporal and spatial factors of samples taken from patients during the control and pre-elimination phases (1993–2007 and 2008–2011, respectively) in southern Mexico.

## 2. Materials and Methods

All subjects were informed of the nature of the study and gave their consent before being included. The protocol was conducted in accordance with the Declaration of Helsinki and was approved by the Ethics Committee of the National Institute of Public Health of Mexico (project CI1042).

The area under study is located in Jurisdiction VII of the state of Chiapas, on the Pacific side of Mexico and very close to the border with Guatemala. In this tropical area, comprising ~4644 km^2^ and ranging from sea level to an altitude of ~4000 m, an elevated number of malaria cases were recorded in the 1990s. Subsequently, the number of cases decreased, as did the number of rural communities affected. Although in 2005 the level of infection reached a low level of 167 cases, it increased to 657 in 2006, probably due in large part to the conditions left by hurricane Stan in 2005. From 2009 on, a significant decline took place, likely because of the implementation of control measures [22]. In this Jurisdiction, malaria cases were between 1645 and 167 annually during the control phase (up to 2007), and have ranged from 657 to 101 annual cases during the pre-elimination phase (from 2008 to the present).

### 2.1. Plasmodium vivax-Infected Blood Samples

For symptomatic patients, malaria diagnoses have for many years been carried out in the laboratory facility at the Regional Center for Public Health Research (CRISP-INSP), as described previously [23]. Blood samples testing positive for *P. vivax* from 1993–2011 were analyzed in the current study [22,24,25,26,27]. The number of samples to study were selected by convenience. Since the available *P. vivax*-infected blood samples were few from 1993 to 2001, all of them were presently included. To reduce bias, samples from 2002 to 2011 were selected from different months of each year. A total of 116 samples, from 1993 to 2000 and 2009 to 2011, were dripped on filter paper and dried, then kept in the dark in a freezer. From 2002 to 2007, the samples were of fresh blood preserved in liquid nitrogen. In 2001 and 2008, they were comprised of both types (fresh and dried blood). Apart from the 116 dry blood samples, there were a total of 101 fresh blood samples.

### 2.2. Gene Amplification and Sequencing

Whole DNA was extracted from *P. vivax*-infected blood samples with the QIAamp^®^ DNA Blood Mini kit (Qiagen, Germantown, MD, USA), as specified by the manufacturer. The gene fragment *P. vivax pvmsp1_42_* was amplified with primers: pvmsp1_42F1 5′-GCCGAGGACTACGACAAAG-3′ and pvmsp1_42R1 5′- CAAGCTTAGGAAGCTGGAGG-3′ [27]. Each PCR reaction included the following: 1X Colorless GoTaq^®^ Flexi Buffer (Promega, Madison, WI, USA), 1 mM MgCl_2_, 0.2 mM dNTPs (Invitrogen, Carlsbad, CA, USA), 10 pM of each primer, 1.25 U GoTaq^®^ DNA Polymerase (Promega, Madison, WI, USA), and 1.5–4 μL of template DNA to constitute a final volume of 50 μL. The PCR conditions began with denaturation at 95 °C for 5 min, followed by 35 cycles: denaturation at 94 °C for 60 s, alignment at 60 °C for 60 s, and extension at 72 °C for 7 s. A final extension was carried out at 72 °C for 10 min. The correct molecular size and specificity of the amplified DNA fragments were verified by electrophoresis in 1% agarose gel. For visualization, the gel with 0.2 μg/mL of ethidium bromide was observed under ultraviolet light in a transilluminator.

Some samples that did not amplify or gave a very low DNA concentration were re-amplified with primers pvmsp1_42F1 and pvmsp1_42R3: 5′ CCCTCAAGAGGGTCAGA 3′ by employing the aforementioned conditions and 1–2 μL of the first PCR product. All PCR reactions were performed in a T100 Thermal Cycler^TM^ (Bio-Rad, Hercules, CA, USA).

The amplified samples displaying a single band with the correct molecular size were purified with the MinElute^®^ PCR Purification Kit (Qiagen, Germantown, MD, USA), according to the manufacturer’s instructions. The purified DNA products were sequenced by the Sanger method in Macrogen, Inc. (Geumcheon-qu, Seoul, Republic of Korea). The nucleotide sequences were reviewed manually with the BioEdit Sequencing Alignment Editor v5.0.9 [28] and aligned with Clustal W [29] by using the Sal-I sequence (GenBank: XM_001614792) as the reference. The consensus sequences were deposited in the NBCI GenBank database with the access numbers of OL411675–OL411837.

### 2.3. Data Analysis

Genetic analysis. The total number of mutations (M), segregating sites (S), and haplotypes (H), as well as the haplotype diversity (Hd), nucleotide diversity (π), and genetic diversity (θ) were determined on dnaSP v5.1 software [30].

Recombination. The minimum number of recombination events detects recombination in the history of a sample [31]. The linkage disequilibrium (LD) is calculated by finding the coefficient of correlation (R) between a pair of loci, then squaring the value (R^2^) [32]. These parameters were computed on the dnaSP v5.1 program (29). The range of R^2^ is 0 to 1, which indicates whether a given locus is incomplete linkage equilibrium or complete LD, respectively.

Natural selection. Natural selection was examined with two approaches. Firstly, the ratio of the fixed substitution rate between the number of substitutions of non-synonymous nucleotides (*dN*) versus synonymous ones (*dS*)—expressed as *dN/dS*—was established on MEGA v6.0 software [33], utilizing the joint maximum likelihood reconstruction of ancestral states based on the Muse-Gaut [34] and Felsenstein codon substitution models [35]. Secondly, to assess the neutral theory of evolution, Tajima’s D test [36] was conducted on the dnaSP v5.1 program (29). This test scrutinizes the difference between two measurements of genetic diversity: the average number of pairwise differences between nucleotides and the number of segregating sites. Positive values of Tajima’s D suggest an excess of common variation in a region, which may be indicative of balancing selection or a contraction of the parasite population. Negative values reveal an excess of rare variations, consistent with population growth.

Haplotype networks. To determine the genealogical relationships between the *pvmsp1_42_* haplotypes, haplotype changes were evaluated over time to define haplogroups, which are haplotype networks constructed with TCS v1.21 software using sequences from the entire time under study and by specific time periods [37], as reported previously [22].

Geographic assessment. To examine their geographic pattern and possible changes over time, haplotypes were mapped according to their local origin with ArcView v3 software [38].

B-cell epitopes. With the BepiPred B-cell predictor server, B-cell epitopes were predicted in PvMSP1_42_ amino acid sequences [39], selecting the epitope prediction with ≥12 consecutive amino acids and 85% specificity: http://ailab-projects1.ist.psu.edu:8080/bcpred/ (accessed on 1 October 2021).

SplitsTree analysis. To explore the overall relationship between haplotypes in southern Mexico and worldwide, homolog sequences of *pvmsp1_42_* were downloaded from NCBI (https://www.ncbi.nlm.nih.gov/ (accessed on 7 May 2021); Table S1) [18,19,20,21,40,41,42,43,44,45,46,47,48]. The SplitsTree analysis was performed with the NeighborNet method on the SplitsTree v4.14.6 program [49]. This is based on haplotype networks and phylogenetic trees and allows for the construction of phylogenetic inferences. The best-fitting nucleotide substitution was GTR, revealing some invariant sites and the gamma distribution rates (I + G), obtained on JModeltest2 software v2.1.10 [50]. The sequence of *Plasmodium cynomolgi* (GenBank: AY869723) served as an outgroup. Supplementary Table S1 shows the groups of sequences acquired from NCBI and employed in the analysis.

## 3. Results

### 3.1. Pvmsp1_42_ Polymorphism and Evaluation of Nucleotide and Haplotype Diversity

The processing of 217 samples afforded 163 *msp1_42_* sequences with a single nucleotide sequence and 936 base pairs (comprising nucleotides 4149–5085 or codons 1384–1695). None of the sequences were similar to the Sal-I strain (XM_001614792.1) [51]. In addition, 26 sequences exhibited double peaks in both forward and reverse pherograms (the other samples did not amplify). Pherograms with double peaks were considered to be multiple genotype infections (MGIs), because double peaks occurred at two or more nucleotide positions in the polymorphic region of *pvmsp1_33_*, while the *pvmsp1_19_* fragment was conserved. Figure S1 illustrates the yearly distribution of the samples that did or did not amplify. Thirty-five single sequences previously reported from the same geographic region (2006–2007) [27] were included in the current analysis, thus constituting a total of 198 sequences. In *pvmsp1_33_*, 57 polymorphic sites and 64 mutations were identified (nucleotides 4093–4917 or codons 1364–1639), as were 7 synonymous substitutions (codons: 1476, acc→acg; 1509, gaa→gag; 1532, agt→agc; 1533, ctg→ttg; 1538, cca→ccg; 1564, gtc→gtt; and 1570, ctg→ctt) and 57 nonsynonymous substitutions (Table S2). On the other hand, *pvmsp1_19_* (nucleotides 4918–5085 or codons 1640–1695) was conserved and similar to the Sal-I sequence.

The nucleotide (π) and genetic diversity (θ) of the 198 single nucleotide sequences were 0.0219 and 0.0104, respectively. The haplotype diversity was 0.802 ± 0.014, finding 17 haplotypes. The most common haplotypes were h1, h4, h5, and h9, found at 31.8%, 21.2%, 14.6%, and 18.6%, respectively. For these sequences, a minimum number of 12 recombination events was estimated, with a correlation coefficient index (R^2^) of 0.332 (LD = R^2^; Table 1). The *dN/dS* and Tajima’s D values for *pvmsp1_42_* were positive, being 1.108 (*p* > 0.05) and 2.650 (*p* < 0.05), respectively (Table 1).

### 3.2. Temporal Analysis of pvmsp1_42_ Nucleotide Diversity, Recombination, and Natural Selection

**Figure 1:**
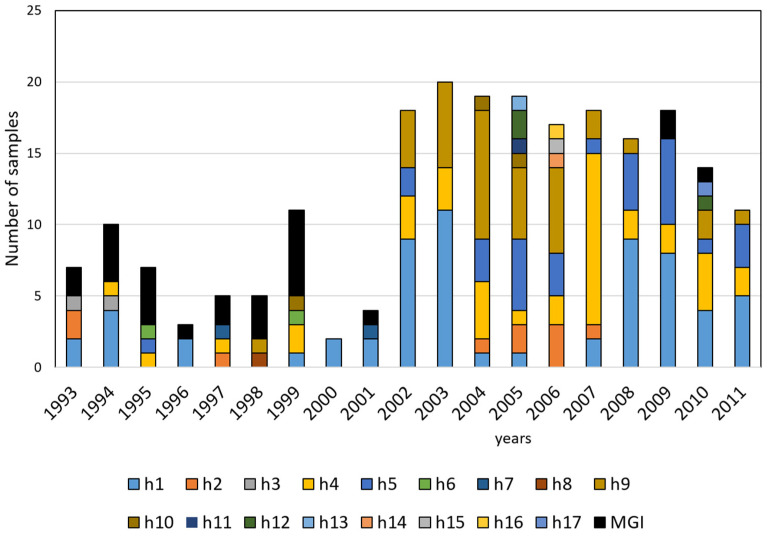
*P. vivax msp1_42_* haplotypes detected in samples from each year. Bars portray single sequences and multiple genotype infections (MGI) from southern Mexico. From 2002–2011, the proportion of the most common haplotypes varied over time.

Of 26 multiple genotype infections encountered presently, there were 22 (84.6%) in samples from the 1990s (Figure 1), and 29–75% of them in any particular year. In contrast, only one of these multiple genotype infections was discovered in the samples from 2001, two in 2009, and one in 2010. A comparison of the proportion of haplotypes was made for 2002–2011, as more than 10 single sequences were obtained per year. The proportion of the distinct haplotypes was similar in samples from 2002 and 2003. In the following years (2004–2007), however, h1 was observed in a lower proportion in those samples. The proportion of haplotypes was significantly different between samples from 2003 and 2004 (Pearson’s chi-squared value (*χ*^2^(5) = 14.5; *p* = 0.015)) and between samples from 2006 and 2007 (*χ*^2^(7) = 16.1; *p* = 0.024). H4 was detected in most of the samples in 2007. During the next four years (2008–2011), the haplotype pattern was similar to that observed in samples from 2002–2003, except for the scant number of parasites containing h9 (as in 2007). The comparison of samples from 2007 and 2008 showed a significant difference in the proportion of haplotypes (*χ*^2^(4) = 14.6; *p* = 0.005; Figure 1).

The *pvmsp1_42_* sequences were grouped into four consecutive time periods. For 1993–2001 samples, only 31 single sequences were obtained and were considered as one group that existed during the years of highest transmission. As this group included many years and few sequences, quantitative outcomes were not analyzed or compared to the other periods, all of which involved similar number of years and more than 50 *P. vivax* sequences: 2002–2004 (*n* = 57), 2005–2007 (*n* = 54), and 2008–2011 (*n* = 56). There was a subtle variation in nucleotide and genetic diversity between samples of these periods, finding *π* and θ values ranging from 0.0205–0.0210 and 0.0111–0.0126, respectively. In contrast, the R^2^ index of LD increased gradually in samples from 2002–2004 to 2008–2011, and the *dN/dS* and Tajima’s D values were positive. In samples from 2008–2011, Tajima’s D values were positive, although *dN/dS* values were not significant (Table 1).

### 3.3. Haplotype Network, Temporal Changes, and Haplogroups

**Figure 2:**
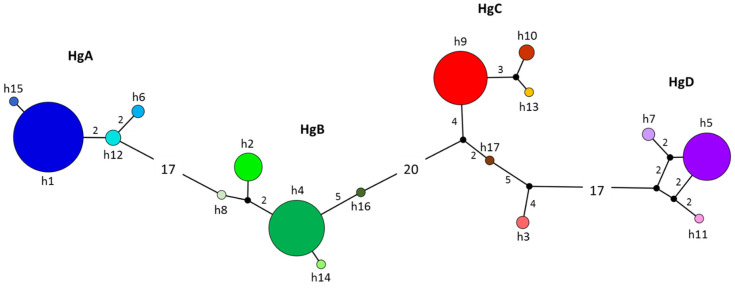
The haplotype network of *P. vivax msp1_42_* from 1993–2011 in southern Mexico. Each color corresponds to one haplotype. The number of samples containing each haplotype is indicated by the size of the circle. Black dots represent existing or extinct haplotypes not found in the samples. The number of mutational steps between haplotypes, if higher than one, is denoted. Four haplogroups were formed (HgA, HgB, HgC, and HgD). In the 198 sequences analyzed, haplotypes h1, h4, h5, and h9 were the most common, found at 31.8%, 21.2%, 14.6%, and 18.7%, respectively. *Pvmsp1_42_* haplotypes displayed a similar pattern of colors as in Figure 1.

The haplotype network demonstrates that the *P. vivax msp1_42_* haplotypes of southern Mexico are separated by 1 to 75 mutational steps. When divided into four haplogroups (Hg: A, B, C, and D), they were visibly separated from each other by 17 to 20 mutational steps. Each Hg comprised at least one high-frequency haplotype and other closely-related low-frequency ones (Figure 2). HgA included the high-frequency haplotype h1 and three low-frequency haplotypes (h12>h6>h15) separated by two, one, and four mutational steps from h1, respectively. In HgB, the high-frequency haplotype h4 was followed by h2 and three low-frequency haplotypes (h8, h14, and h16), separated by three, three, one, and five mutational steps from h4, respectively. HgC contained the high-frequency haplotype h9 and three low-frequency haplotypes (h10, h17, and h13), which were separated by four, six, and four mutational steps from h9. Haplotype h3, detected in two parasites from 1993 and 1994, was classified as HgC even though it was separated by 15 mutational steps from h9. HgD consisted of the high-frequency haplotype h5 and two low frequency haplotypes (h7 and h11), separated by three and four mutational steps from h5 (Figure 2). The temporal network showed that the high-frequency haplotypes from each Hg were encountered in sequences from all time periods, although changes in the proportion were observed in certain periods within 2002–2011. Some of the low-frequency haplotypes of each Hg were found in different periods (h12, h2, and h10), while others were detected in only one isolate (Figure S2).

### 3.4. Spatiotemporal Distribution of the Haplogroups in Southern Mexico

**Figure 3:**
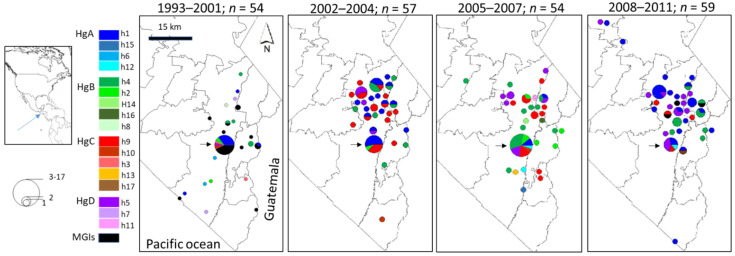
Geographic distribution of *pvmsp1_42_* haplotypes during four time periods from 1993–2011. The size of the circles is proportional to the number of isolates from each rural area and Tapachula City. Haplogroups are illustrated in the same colors and haplotypes in the same shades as in Figure 2. The spatial pattern of haplogroups varied from one period to another. The multiple genotype infections (MGIs) detected in 1993–2001 displayed a scattered distributed in the region. The largest circle on each map corresponds to Tapachula City (short arrow). A significant difference in the proportion of haplotypes existed when comparing samples from 2002 to 2004 and 2005 to 2007 [*χ*^2^ (11) = 27.2781; *p* = 0.004], 2005 to 2007 and 200 to 2011 [*χ*^2^ (12) = 38.4027; *p* = 0.000], and 2002 to 2004 and 2008 to 2011 [*χ*^2^ (7) = 18.5703; *p* = 0.010].

The geographic pattern of haplogroups varied with time (Figure 3). Most sequences came from the municipality of Tapachula and its surrounding areas. The high-frequency haplotypes in each sample/period were detected in Tapachula City. In the 1990s, most sequences were from the city and fewer came from the outlying hilly and coastal regions. From 2002 on, most parasite sequences came from a combination of the outlying hilly areas and the city.

All haplogroups and the haplotypes in each group were scattered across the geographic area (Figure 3). In 1993–2001, haplotypes of the different haplogroups were present in the city and rural areas. Members of HgA, HgB, HgC, and HgD were found in Tapachula City in sequences from all time periods. HgA haplotypes were identified in 7 rural areas in sequences from 2002 to 2004, 4 in 2005–2007, and 18 in 2008–2011. HgB constituents were encountered in 8 rural areas in 2002–2004, 16 in 2005–2007, and 7 in 2008–2011. HgC members were evidenced in 14 rural areas in 2002–2004, 8 in 2005–2007, and 3 in 2008–2011. Lastly, h5 in HgD was discovered in 3 rural areas in 2002–2004, 6 in 2005–2007, and 10 in 2008–2011. The city was the site with the highest number of sequences. It also showed the greatest variation in the composition of each Hg when comparing the sequences from 2002 to 2004, 2005 to 2007, and 2008 to 2011 (Figure 3).

### 3.5. Linear B-Cell Epitopes

**Figure 4:**
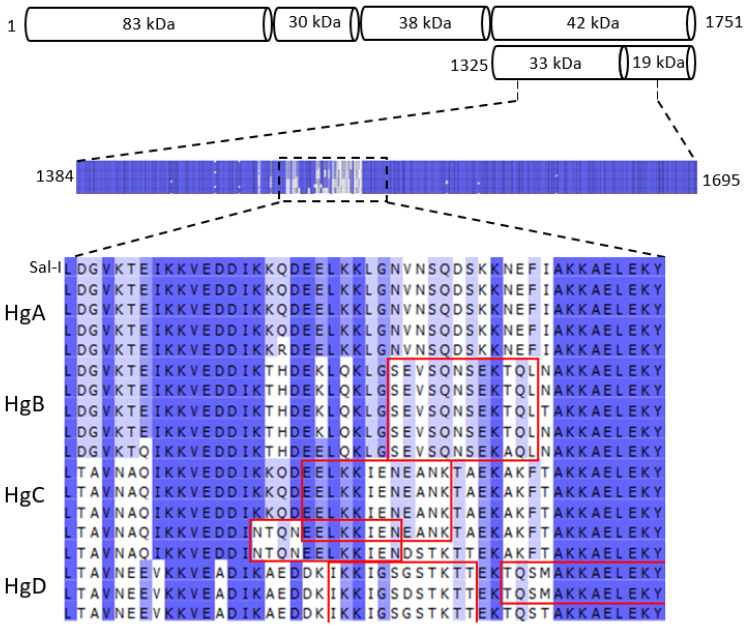
The *P. vivax* MSP1-42 kDa highly variable peptide segment (amino acids 1489–1536) and the predicted B-cell epitopes. Different B-cell epitopes were predicted for each haplogroup with the Bcpred web server (in the red boxes). The epitope prediction of ≥12 consecutive amino acids was obtained by using 85% specificity. The Sal-I sequence was similar to h1 from southern Mexico.

In the polymorphic amino acid sequences, haplogroup-specific B epitopes were predicted (Figure 4). In HgA, one amino acid changed from a polar to a positive charge (Q1506R in h15). This Hg was similar to the Sal I sequence. In the highly variable region, the peptide SEVSQNSEKTQL in HgB and two peptides (EELKKIENEANK and NTQNEELKKIEN) in HgC were predicted to participate in B-cell epitopes. In HgD, the peptides IKKIGSGSTKTT and TQSMAKKAELEKY were predicted to participate in B-cell epitopes. Additionally, three peptides from the semi-conserved carboxyl region of the 33 kDa fragment between codons 1668 and 1730 (EEYKKSEKKNEV, NCQLEKKEAEIT, and SKLIKENESKEI) were predicted to participate in B-cell epitopes.

### 3.6. SplitsTree Analysis

**Figure 5:**
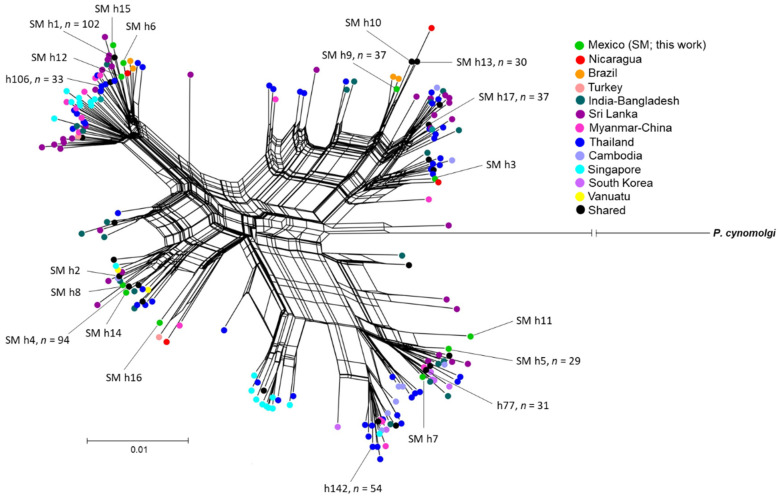
SplitsTree analysis of the global distribution of *P. vivax msp1_42_*. The four main clusters found with the NeighborNet method resembled haplogroups A, B, C, and D (established by the haplotype network). Only the most common worldwide haplotypes (≥29) and those from this study are portrayed. *P. cynomolgi* served as the outgroup. The bar scale denotes the nucleotide substitution per site.

*Pvmsp1_42_* sequences from parasites in southern Mexico were compared to 631 homologous sequences from other geographic regions of the world. In 829 sequences, 117 segregating sites and 206 haplotypes were observed. The SplitsTree analysis of all haplotypes showed four main clusters with distinct assemblies and branches that contained parasites from the different parts of the world (Figure 5). Eleven of the seventeen haplotypes were exclusive to southern Mexico, including all those in HgD. For HgC, the high-frequency h9 and low-frequency h3 were exclusive to southern Mexico, whereas low-frequency h10 and h13 were shared with Nicaragua, and low-frequency h17 was detected at various sites (e.g., South Korea). HgA was closely related to the Sal-I sequence, and its high-frequency h1 was found in southern Mexico and Nicaragua, while its low-frequency haplotypes (h6, h12, and h15) were exclusive to southern Mexico. The most common haplotypes of HgB, being high-frequency h4 and low-frequency h2, were shared with Brazil, Turkey, and South Korea. One of them was also discovered in Thailand. The low-frequency haplotypes of HgB (h8, h14, and h16) were exclusive to southern Mexico. Worldwide, the three most common haplotypes were identified in Asian parasites—h106 and h142 from Thailand and h77 in Sri Lanka and South Korea (Figure 5). Based on the complex network, no particular structure of the parasite population existed at the global level. *Plasmodium cynomolgi* was rooted in between the HgC and HgD networks (Figure 5).

## 4. Discussion

According to the genetic analysis of *pvmsp1_42_*, the *P. vivax* population seems to have evolved locally, probably in Mesoamerica. The moderate level of nucleotide and haplotype diversity allowed for the evaluation of transmission dynamics during the control and pre-elimination phases (2002–2011). Despite the lower number of samples from the 1990s, the multiple genotype infections were notably high, which concurs with the high malaria transmission in those years. Although there were only subtle variations in the nucleotide and genetic diversity for *pvmsp1_42_*, the haplotype diversity diminished and the R^2^ index of LD increased in samples from the pre-elimination period (samples from 2008 to 2011) compared to those periods from the control phase (samples from 2002 to 2004 and 2005 to 2007). Tajima’s D value was significantly high only for the samples from 2008 to 2011.

These results indicate that *P. vivax* from southern Mexico probably underwent recombination and diversification processes, leading to the formation of several *pvmsp1_42_* haplogroups. Each Hg consisted of one high-frequency haplotype and other low-frequency ones. The current results resemble the published data obtained using microsatellites [52], the *pvmsp1* segment icb5-6 [53], and *pvama1* [22], as well as genomic research [54]. The high-frequency *P. vivax* haplotypes observed for *pvmsp1_42_* (h1, h4, h5, and h9) represent 86.3% of the 198 single sequences (the entire sample). The four haplogroups formed for *pvmsp1_42_* were detected in a dispersed pattern in the region under study, suggesting widespread circulation of the main haplotypes—likely by both human and parasite factors. For example, human activities requiring mobility across rural areas or municipalities and episodes of relapse would seem to have affected haplotype circulation. The most common haplotypes persisted over time, which must have played an important role in the similar nucleotide and genetic diversity values estimated for the different time periods.

A moderate diversity of *pvmsp1_42_* in southern Mexico was evidenced by a previous report. The overall nucleotide diversity calculated in the current contribution (198 sequences from 1993 to 2011) is similar to that found for 35 sequences from 2006 to 2007 [27]. This might be due to hurricane Stan, which created conditions favoring the transmission of endemic and relapsing *P. vivax* haplotypes in the years immediately after 2005 [55]. A greater number of low-frequency haplotypes were identified when the number of *P. vivax* cases was higher. In the low endemic area of southern Mexico, the presence of multiple genotype infections in the human host may have favored the recombination of different parasite haplotypes in the mosquito vector [56,57]. However, the vector specificity previously observed in the region could have restricted recombination for certain parasite haplotypes [52,58]. The number of isolates exhibiting *pvmsp1_42_* single sequences in the 1990s was low, perhaps impeded by the detection of other low-frequency alleles. This issue cannot be resolved in the current study. Importantly, the analysis of *pvama1_II_* in infected blood samples from the 1990s showed multiple genotype infections consisting of high- and low-frequency haplotypes [22].

The R^2^ index of LD and Tajima’s D values rose as transmission decreased, suggesting a contraction in the parasite population during the years of the pre-elimination phase (2008–2011). Not only are these results congruent with previous findings using *Pvama1_II_* [22], but they are also supported by the lack of malaria outbreaks after 2007, as well as the sensitivity of *P. vivax* to treatment based on chloroquine and primaquine [59]. The comparison of 2004 and 2013–2015 in a highly endemic region of Myanmar, contrarily, revealed a reduction in the number of malaria cases, a variation in the frequency of genetic clusters of *pvmsp1*-icb5-6, and an increase in the number of haplotypes and the values for recombination events [60]. Although Myanmar has set a target date of 2030 for the elimination of malaria, it faces a complex scenario of haplotype diversity and drug-resistance.

Malaria transmission in southern Mexico underwent a slow decline in the 1990s, reflected in a lower number of malaria cases. Since the year 2000, this decrease has continued as a result of intensive vector control campaigns, the active detection of malaria cases, and the administration of chloroquine and primaquine treatment schemes [2]. During the sample collection, the treatment for *P. vivax* involved monthly and intermittent single doses of chloroquine and primaquine. It was estimated that about 50% of the patients had at least one relapse of malaria [59]. Under that scenario, endemic/relapsing haplotypes became highly adapted and persisted overtime.

No differences existed in nucleotide and genetic diversity between the sequences from control periods (2002–2004 and 2005–2007) and the sequences from the pre-elimination period (2008–2011). However, variation in the proportion of some haplotypes and haplogroups was evident from one year to another. Although immunogenicity and natural antibody responses against PvMSP1_19_ [61,62] and PvMSP1_42_ [13,63] have been reported in *P. vivax*-infected patients. The PvMSP1_33_ fragment, on the other hand, is highly variable compared to PvMSP1_19_ [18,19,20,21,43,44,64], and is presumably involved in the evasion of the immune responses [19]. Temporal changes in the proportion of the haplogroups might have been the result of balancing selection acting on the polymorphic fragment PvMSP1_33_, an idea supported by the excess of non-synonymous mutations and positive *dN/dS* values.

Additionally, the four haplogroups are different from the B-cell epitopes predicted by the BepiPred program. Under these conditions, haplogroup- or haplotype-specific antibody responses may be generated in the host and contribute to the “immature” natural responses, as proposed by Wickramarachchi et al. [14]. In low-transmission regions, the half-life of the immune response following a *P. vivax* infection is suggested to be less than six months [27,62]. In the absence of frequent blood infections, therefore, immunity to the blood stages of the parasite becomes deficient during the transmission season. In one study, some immune serum samples from patients were reactive to only one of the two haplotypes of PvMSP1_33_ (the Sal-I and Belem strains) [63]. Further research at the community level might be necessary to decipher whether or not antibody responses against distinct PvMSP1_33_ immunotypes can cause haplotype rotation in hypo-endemic settings. Otherwise, genetic drift could have contributed to haplotype or haplogroup fluctuations over time. Many *pvmsp1_42_* haplotypes were exclusive to southern Mexico or Mesoamerica, which indicates that this gene fragment might be instrumental in distinguishing native *P. vivax* strains from those introduced from other regions.

## 5. Conclusions

*Pvmsp1_42_* was adequate to estimate genetic changes in sequences from the control and pre-elimination phases. Unlike samples from the control phase, those from the pre-elimination period were characterized by an insignificant rate of multiple genotype infections, a reduction in haplotype diversity, and an increase in LD and Tajima’s D values—indicative of a contraction in the parasite population. All four haplogroups of the present study exhibited specific B-cell epitopes, and all displayed a scattered pattern of dispersion in the region, with some fluctuations over time. The results suggest that the elimination of the malaria parasite will require the maintenance of malaria cases at a low level until transmission is interrupted. PvMSP1_42_ is a good candidate for molecular surveillance to evaluate anti-malaria programs. Further research is indispensable to elucidate whether or not the presumed fluctuations of the haplogroups in PvMSP1_42_ were due to immune evasion.

## Figures and Tables

**Table 1:** Genetic parameters of *P. vivax msp1_42_* from southern Mexico, analyzed by time-periods.

Period (Years)	Genetic Diversity							Recombination		Natural Selection				
	N	M	S	H	Hd (SD)	π (SD)	θ (SD)	Rm	R^2^	*dN*	*dS*	*dN/dS*	Z-Test *p* Value	Tajima’s D
1993–2001	31	60	54	10	0.798 (0.062)	0.0196 (0.002)	0.0144 (0.002)	8	0.350	38	6	0.556	0.290	0.831
2002–2004	57	57	51	6	0.727 (0.030)	0.0209 (0.001)	0.0118 (0.001)	7	0.357	37	7	1.151	0.126	1.981
2005–2007	54	61	54	12	0.834 (0.027)	0.0210 (0.001)	0.0126 (0.001)	10	0.393	37	8	1.282	0.101	1.810
2008–2011	56	54	48	6	0.697 (0.041)	0.0205 (0.001)	0.0111 (0.001)	7	0.420	36	5	1.217	0.113	2.161 *
Full period	198	64	57	17	0.802 (0.014)	0.0219 (0.001)	0.0104 (0.001)	12	0.332	37	7	1.108	0.135	2.650 *

N, number of sequences; M, total number of mutations; S, segregating sites; H, number of haplotypes; Hd, haplotype diversity; π, nucleotide diversity; θ, genetic diversity; Rm, minimal number of recombination events; LD, linkage disequilibrium, expressed as the R^2^ index; *dN*, nonsynonymous substitutions; *dS*, synonymous substitutions. * *p* < 0.05.

## Data Availability

All relevant data is contained within the article or Supplementary Materials.

## Supplementary Materials

Figures S1 and S2 and Tables S1 and S2 are available online at: https://www.mdpi.com/article/10.3390/microorganisms10010186/s1. Figure S1: *P. vivax* samples from southern Mexico, 1993-2011. Figure S2: Temporal haplotype distribution of *Plasmodium vivax pvmsp1_42_* in southern Mexico. Table S1: Origin and accession numbers of the *pvmsp1_42_* sequences obtained from NCBI at https://www.ncbi.nlm.nih.gov/, accessed on 5 January 2022. Table S2: Haplotypes and amino acid substitutions for PvMSP1_42_ kDa parasites in southern Mexico.
