# Supplementary material for: Spatiotemporal Changes in Plasmodium vivax msp142 Haplotypes in Southern Mexico: From the Control to the Pre-Elimination Phase
Source: Microorganisms. 2022 Jan 15;10(1):186. doi: 10.3390/microorganisms10010186 (PMC8779127; doi:10.3390/microorganisms10010186)
Supplement: Supplementary file 1 [file microorganisms-10-00186-s001.zip › suplementary materials/Table S1.pdf]

Table S1. Origin and accession numbers of the *pvmsp1₄₂* sequences obtained from NCBI; https://www.ncbi.nlm.nih.gov/ (accessed on 28 December 2021).

GenBank accession number	Number of sequences	Region	Country	References
KR871926 - KR872017	92	Central America	Nicaragua	Gutiérrez et al., 2016[40]
AF435593<br>AF435594<br>AF435622- AF435625<br>AF435627<br>AF435629- AF435631<br>AF199405- AF199407	13	South America	Brazil	Putaporntip et al., 2002[41]<br>Putaporntip et al., 2000[42]
AB564559 - AB564588	30	Middle East	Turkey	Zeyrek et al., 2010[43]
EU430452 - EU430479<br>AF435616 - AF435620<br>KF612323<br>AF435639	35	South of Asia	India - Bangladesh	Thakur et al., 2008[44]<br>Sheikh et al., 2014[45]<br>Putaporntip et al., 2002[41]
GU175174 - GU175268<br>AJ292349 - AJ292359	106	South of Asia	Sri Lanka	Dias et al., 2011[19]<br>Manamperi et al., 2016 unpublished
AF199393 - AF199404<br>AF199408 - AF199410<br>AF435595 - AF435599<br>AF435601 - AF435615<br>GQ890872 - GQ891041<br>GQ912337	206	Southern Asia	Thailand	Putaporntip et al., 2000[42]<br>Putaporntip et al., 2002[41]<br>Jongwutiwes et al., 2010[46]
JX461286 - JX461295<br>JX461300 - JX461310<br>JX461312 - JX461317<br>JX461319 - JX461333<br>JX461297<br>JX461298	44	Southern Asia	Cambodia	Parobek et al., 2014[20]
GU971656 - GU971705	50	Southern Asia	Singapore	Ng et al., 2010[47]
JX490129 - JX490156<br>JX993754<br>JX993755	30	Southern Asia	Myanmar-China	Zhou et al., 2017[21]
HQ171934 - HQ171941<br>AF435635 - AF435638<br>JQ446312 - JQ446322	23	East Asia	South Korea	Han et al., 2011[48]<br>Putaporntip et al., 2002[41]<br>Kang et al., 2012[18]
AF435632<br>AF435634	2	Oceania	Vanuatu	Putaporntip et al., 2002[41]
