# Supplementary figures and images for: Spatiotemporal Changes in Plasmodium vivax msp142 Haplotypes in Southern Mexico: From the Control to the Pre-Elimination Phase
Source: Microorganisms. 2022 Jan 15;10(1):186. doi: 10.3390/microorganisms10010186 (PMC8779127; doi:10.3390/microorganisms10010186)

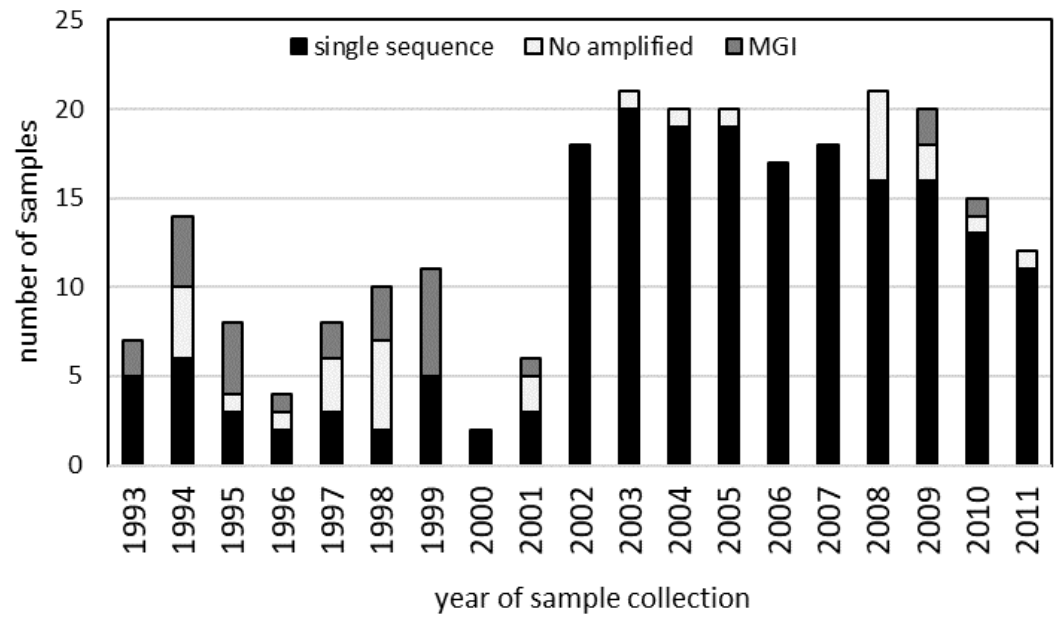

**Figure S1.** *P. vivax* samples from southern Mexico, 1993-2011.

Supplement: Supplementary file 1 [file microorganisms-10-00186-s001.zip › suplementary materials/microorganisms-1551946 - supplFigS1.pdf]

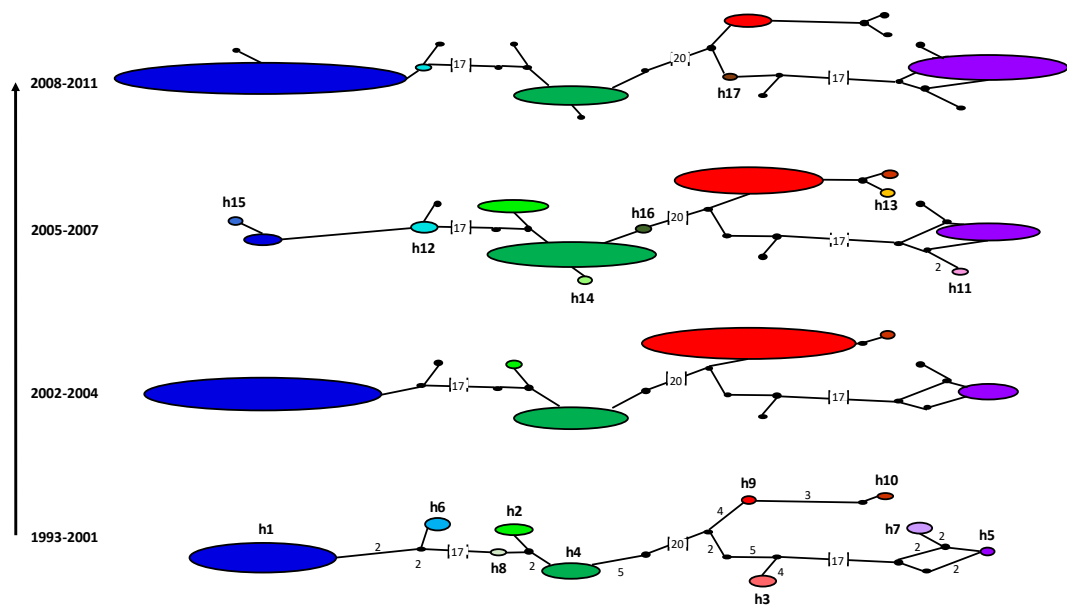

**Figure S2.** Temporal haplotype distribution of *Plasmodium vivax* pvmsp142 in southern Mexico.

Supplement: Supplementary file 1 [file microorganisms-10-00186-s001.zip › suplementary materials/microorganisms-1551946 - supplFigS2.pdf]
